# Phylogenetic and Phylodynamic Analyses of Human Metapneumovirus in Buenos Aires (Argentina) for a Three-Year Period (2009–2011)

**DOI:** 10.1371/journal.pone.0063070

**Published:** 2013-04-30

**Authors:** Ana Julia Velez Rueda, Alicia Susana Mistchenko, Mariana Viegas

**Affiliations:** 1 Laboratorio de Virología, Hospital de Niños “Dr. Ricardo Gutiérrez”, Ciudad Autónoma de Buenos Aires, Argentina; 2 Comisión de Investigaciones Científicas (CIC), La Plata, Provincia de Buenos Aires, Argentina; 3 Consejo Nacional de Investigaciones Científicas y Técnicas (CONICET), Ciudad Autónoma de Buenos Aires, Argentina; University of Texas Medical Branch, United States of America

## Abstract

Human metapneumovirus, which belongs to the *Paramyxoviridae* family and has been classified as a member of the *Pneumovirus* genus, is genetically and clinically similar to other family members such as human respiratory syncytial virus. A total of 1146 nasopharyngeal aspirates from pediatric patients with moderate and severe acute lower respiratory tract infections, hospitalized at the Ricardo Gutierrez Childreńs Hospital (Buenos Aires, Argentina), were tested by real time RT-PCR for human metapneumovirus. Results showed that 168 (14.65%) were positive. Thirty-six of these 168 samples were randomly selected to characterize positive cases molecularly. The phylogenetic analysis of the sequences of the G and F genes showed that genotypes A2 and B2 cocirculated during 2009 and 2010 and that only genotype A2 circulated in 2011 in Argentina. Genotype A2 prevailed during the study period, a fact supported by a higher effective population size (Neτ) and higher diversity as compared to that of genotype B2 (10.9% (SE 1.3%) vs. 1.7% (SE 0.4%), respectively). The phylogeographic analysis of the G protein gene sequences showed that this virus has no geographical restrictions and can travel globally harbored in hosts. The selection pressure analysis of the F protein showed that although this protein has regions with polymorphisms, it has vast structural and functional constraints. In addition, the predicted B-linear epitopes and the sites recognized by previously described monoclonal antibodies were conserved in all Argentine sequences. This points out this protein as a potential candidate to be the target of future humanized antibodies or vaccines.

## Introduction

Human metapneumovirus (HMPV), which belongs to the *Paramyxoviridae* family and has been classified as a member of the *Pneumovirus* genus [1], is genetically and clinically similar to other family members such as human respiratory syncytial virus (HRSV) and human parainfluenza type 3 virus.

The HMPV genome is approximately 13.3 Kb in length and contains eight genes that are ordered 3′-N-P-M-F-M2-SH-G-L-5′ and encode nine different proteins. Three transmembrane glycoproteins protrude from its envelope: the attachment glycoprotein (G), the fusion protein (F) and the small hydrophobic protein (SH) [2]. The G and F proteins present great immunogenicity and can stimulate the production of neutralizing antibodies [3], [4].

HMPV can be classified into two genetic groups (A and B) and each group can be further divided into two genotypes (A1 and A2, and B1 and B2, respectively) [5], [6]. Although this grouping of genotypes is concordant regardless of which gene is studied (G or F), the G gene appears to allow the best discrimination (as observed for HRSV) [6]. The G protein is of particular interest because its variability at nucleotide and amino acid level is greater than of other proteins, both between and within groups and genotypes [1]. Recent publications have shown the possible cocirculation of groups and genotypes and the predominance of one of them within a geographic region [7].

HMPV infections are observed in all age groups, with a high incidence in pediatric patients, being children under 6 months of age the most affected ones. Serological studies have suggested that ∼70% of children are infected with HMPV by the age of 5 years [1]. The nosocomial impact of HMPV is estimated to be as high as that for HRSV [8]. Children infected with HMPV typically present respiratory symptoms clinically indistinguishable from those elicited by HRSV, such as rhinorrhea, fever and cough as upper respiratory symptoms, and asthma exacerbations, bronchiolitis and pneumonia as the severe presentations of acute lower respiratory tract infections (ALRI) [9]. As with HRSV, infants at risk for severe respiratory infections with HMPV are those with congenital heart disease, chronic lung disease, immunocompromised infants and premature children [10], [11].

Taking into account that there is yet no vaccine or prophylactic therapy to prevent HMPV infections, as there is for HRSV (Palivizumab), it is important to recognize the relevance of HMPV as an ALRI etiologic agent in children under one year of age and in high-risk patients.

In this study, we aimed to characterize the HMPV strains which produced moderate and severe ALRI in hospitalized children in Buenos Aires (Argentina) during a three-year period (2009–2011) by an exhaustive analysis of the G and F genes. In addition, based on the molecular analysis, we aimed to describe the global transmission chains of HMPV in order to deepen the general knowledge of the virus genetic background for the future development of potential treatments and/or vaccines.

## Materials and Methods

### Ethics Statement

Written informed consent was obtained from next of skin, caretakers, parents or guardians on the behalf of the minors/children. The study was approved by the Medical Ethic and Research Committees of Dr. Ricardo Gutiérrez Children’s Hospital (IRB N° 07-030). All samples were coded prior to analysis to ensure anonymity, according to the Declaration of Helsinki and the *Habeas Data* law on protection of personal data (Law N° 25326, Argentina).

### Sample Collection

A total of 1146 nasopharyngeal aspirates (NPA) from pediatric patients with moderate and severe ALRI, hospitalized at the Ricardo Gutierrez Childreńs Hospital of Buenos Aires (Argentina) during 2009–2011 were analyzed by real time RT-PCR for HMPV, as previously described [12].

According to the severity of the ALRI, patients were classified into two groups: moderate cases: patients with a diagnosis of bronchiolitis, pneumonitis or pneumonia, no requiring mechanical respiratory assistance; and severe cases: patients with diagnosis of bronchiolitis, pneumonitis and pneumonia or chronic lung disease (high risk patients), requiring intensive care and respiratory support.

The clinical samples with positive diagnosis for HMPV were kept frozen at −70°C until molecular analysis.

### RNA Extraction and Nucleotide Amplification of Partial G and F Genes of HMPV

Nucleic acids were extracted directly from clinical samples with a PureLink viral RNA/DNA minikit (Invitrogen Life Technologies, Carlsbad, CA, USA). RNA was reverse-transcribed and amplified using the Qiagen OneStep RT-PCR kit (Qiagen, GmbH, Hilden, Germany) following the manufacturer’s instructions with the primers for both partial G and F genes, previously described [13].

The retrotranscription and amplification conditions for the G gene were: 50°C for 30 min; 95°C for 15 min; 94°C for 1 min, 59°C for 1 min, 72°C for 2 min for 34 cycles and 72°C for 7 min. For the F gene, the amplification protocol was the same but using an annealing temperature of 50°C and 39 cycles.

### HMPV G and F Gene Sequences

PCR products were electrophoresed in a 1% agarose gel stained with ethidium bromide and purified with the Zymoclean™ Gel DNA Recovery Kit (Applied Biosystems, Foster City, CA, USA). The purified PCR products were sequenced in forward and reverse directions by using the BigDye Terminator v3.1 cycle sequencing kit (Applied Biosystems, Foster City, CA, USA) on the ABI3500 genetic analyzer (Applied Biosystems).

The nucleotide sequences of the G protein gene and the F protein gene were manually edited with BioEdit v7.1.3 [14] and aligned with CLUSTAL W [15]. All these sequences were submitted to GenBank (accession numbers KC210054 to KC210091).

### Phylogenetic Analyses

For the phylogenetic and evolutionary analyses, the G and F gene sequences of HMPV that largely represent the globally circulating strains were downloaded from GenBank (Table S1). Strains CAN83-97_A2_ and CAN75-98_B2_ from Canada (accession numbers AY485253 and AY297748/AY145289, respectively) and strains NL/1/99_B1_, NL/17/00_A2_ and NL/1/94_B2_ from the Netherlands (accession numbers AY304361/AY2960347, AY296021, and AY304362/AY296040, respectively) were used as prototypes of each group and genotype. The most suitable nucleotide substitution model for the Bayesian Markov Chain Monte Carlo (MCMC), Maximum Likelihood (ML) and Neighbor joining (NJ) analyses was selected with MEGA v5.05 [16]. The Bayesian MCMC inference was performed with BEAST v1.7.4 (http: //beast.bio.ed.ac.uk) [17], whereas ML and NJ were performed with MEGA v5.05.

### Evolutionary Rates, Population Dynamics and Phylogeographic Analyses

Nucleotide substitution rates, divergence times, demographic histories and a discrete phylogeographic analysis were estimated from the sequences stamped with date and location using the Bayesian approach with the BEAST v1.7.4 package. The results from BEAST were analyzed using Tracer v1.5 (http: //beast.bio.ed.ac.uk/) to determine the time of the most recent common ancestor (tMRCA) and the nucleotide substitution rates and to plot the demographic histories (Bayesian Skyline Plots (BSP)). The BSP were used to estimate the changes in the population size. These results were also analyzed using Tree-Annotator v1.7.4 to infer a maximum clade credibility (MCC) tree. The trees were visualized and edited with FigTree v1.3.1 (http: //tree.bio.ed.ac.uk/software/figtree). The SREAD (Spatial Phylogenetic Reconstruction of Evolutionary Dynamics) program [18] was used to summarize the discrete phylogeographic analysis in the diffusion dynamic plots and the interactive virtual global animations (KML files), played by Google Earth (http: earth.google.com).

### Molecular Characterization: Adaptive Evolutionary Analysis

Estimates of genetic distances (number of nucleotide or amino acid substitutions per site obtained by averaging all sequence pairs) were used to calculate the divergence between sequences within each group, within genotypes and between years, using MEGA v5.05. Standard error estimates (SE) were obtained by the bootstrap method (1000 replicates). The DNAsp v5 (DNA Sequence Polymorphism) software as used to calculate the nucleotide sequence polymorphisms for both groups and both genes [19].

Natural selection on the HMPV G and F genes was estimated from the ratio of non-synonymous (*dN*) to synonymous (*dS*) substitutions per site (*dN/dS)* at every codon in the alignment and the overall ω = *dN/dS.* The analysis was performed by using the following codon-based ML methods: SLAC [single likelihood ancestor counting], FEL [fixed effects likelihood], IFEL [internal fixed effects likelihood], and REL [random effects likelihood], at the specified significance levels (P = 0.1 and Bayes factor = 50), and using the procedures available in the HyPhy package and accessed through the Datamonkey web server [20], [21].

The potential N-glycosylation sites were predicted with N-Glycosite, using the free public server available on line (http: //www.hiv.lanl.gov/content/sequence/GLYCOSITE/glycosite.html) [22], whereas the O-glycosylation sites were predicted with NetOglyc, using the free public server available on line (http: //www.cbs.dtu.dk/services/NetOGlyc/) [23].

To predict potential B-linear epitopes, the AAP (Amino acid pair antigenicity predictor) and FBCPred (flexible length linear B-cell epitopes predictor) methods [24] (from BCPREDS B-cell epitope prediction server: http: //ailab.cs.iastate.edu/bcpreds/) were used. To perform comparable predictions, 14 epitope lengths were selected with a specificity of 75% and only non-overlapping predicted epitopes were informed.

The amino acid sequences required for the analyses were inferred using the universal genetic code.

## Results

**Figure 1:**
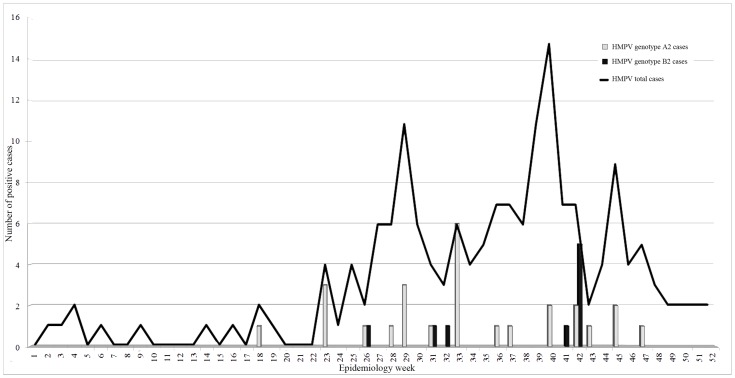
human metapneumovirus annual distribution. The total number of HMPV cases distributed in epidemiology weeks during the study period (2009–2011), are represented by a continuous line. Number of cases of genotype A2 and B2 are represented by gray bars and black bars, respectively.

A total of 168 (14.65%) out of the 1146 NPA tested by real time RT-PCR were positive for HMPV. The annual frequencies were: 21.27% for 2009, 15.38% for 2010 and 10.52% for 2011. Most affected patients were children under 1 year of age (51.78%) and the circulation of HMPV during the study period was highest between epidemiology weeks 32 and 47 (late winter and spring) (Figure 1). The most frequent clinical ALRI presentations were bronchiolitis (53%) and pneumonia (36%).

To characterize positive cases molecularly, 36 out of the 168 positive cases were randomly selected each year of the study. Most selected patients corresponded to pneumonia and high risk patients (63%).

### Phylogenetic Analysis

To typify the Argentine strains, a Bayesian phylogenetic reconstruction was performed with 30 Argentine G sequences and prototype A and B sequences downloaded from GenBank. Similar tree topologies and statistical support were obtained by ML and NJ inferences (data not shown). The analysis showed that 23 sequences clustered with group A and 7 with group B (trees upon request).

**Figure 2:**
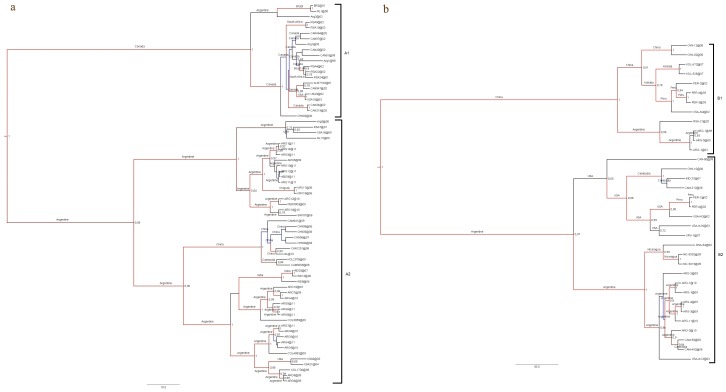
Maximum Clade Credibility trees. Phylogenetic analysis and discrete phylogeographic analysis. The Tamura-Nei 93 model with a discretized gamma distributed among-site rate variation (TN93+G) with four categories was used for all the analyses. Phylogeny was inferred with representative sequences retrieved from GenBank (Tree Coded Names, GenBank accession numbers, genotypes and countries of origin are listed in Table S1). The data set was analyzed assuming a relaxed (uncorrelated lognormal) molecular clock. Bayesian Markov Chain Monte Carlo (MCMC) chains were run for 10 million ngen, 1000 samplefreq and 1000 burnin to reach convergence (expected sample size>200). Only posterior probabilities above 0.7 are shown. The discrete phylogeographic analysis was estimated from the sequences stamped with date and location. Locations of ancestor strains for individual clades are indicated in each clade. **a)** HMPV group A and **b)** HMPV group B.

To define genetic clades and circulation patterns within each group, an exhaustive phylogenetic analysis was performed for each group separately. All the Argentine strains from group A clustered with genotype A2, whereas those from group B clustered with genotype B2 with high posterior probabilities (Figures 2a and 2b). Genotypes A2 and B2 cocirculated during 2009 and 2010, whereas only genotype A2 circulated in 2011. Genotype A2 prevailed during the study period. The same phylogenetic analyses were performed with eight Argentine F sequences and sequences downloaded from GenBank and similar genotype associations were obtained (trees upon request).

The phylogenetic analysis for group A also showed that there were some Argentine strains which remained circulating in our country during the study period supported by their association in the same genetic clade with high posterior probabilities (ARG7 and 10 from 2009, ARG4 from 2010, and ARG5, 6 and 8 from 2011; Figure 2a), and with a maximum mean distance between them of 3.4% (SE 1.1%), denoting microcirculation without evidence of annual alternation of genetic clades. Besides, the association of the Colombian strain COL3859 from 2009 in this genetic clade suggests that there was regional circulation of HMPV, supported by a maximum mean distance between the latter and the previously mentioned Argentine genetic clade of 2.7% (SE 0.9%). On the other hand, there was also an alternation of viral variants during the study period, evidenced by strains from 2009 and 2011 associated in a highly supported single genetic clade (ARG5 from 2009 and ARG1, ARG3, ARG9-12 and ARG14 from 2011) with a maximum mean distance between them of 2.5% (SE 0.9%), and a distance value of 6.7% (SE 1.7%) with Argentine strains from 2010 from a different genetic clade (ARG10 and ARG12) (Figure 2a). However, there was a close association of the Argentine strain ARG12 from 2010 with the Peruvian strain PER9903 from 2009, with a mean distance between them of 1.6% (SE 0.6%), supporting the idea of a regional circulation of the virus in successive outbreaks. Furthermore, a cosmopolitan circulation was denoted with a highly supported association between Argentine strains and other strains from more distant countries such as the USA, India, Cambodia, the Netherlands, and South Africa.

For group B, the phylogenetic analysis revealed that strains closely related to each other and also related to strains previously reported in Cambodia in 2008 circulated in Argentina during 2009–2010 (Figure 2b), with a maximum mean distance between them of 1.5% (SE 0.5%). At the regional level, the maximum mean distance between genotype B2 Uruguayan strains from 2007 and Argentine strains from 2009 and 2010 was 12% (SE 1.8%). However, Nicaraguan strains from 2009 (NIC8902 and NIC9019) were associated with Argentine strains of the same year (ARG2-4) in a well supported genetic clade and with a maximum mean distance between them of 3.4% (SE 1%), supporting the idea of regional movement of viral variants. Strikingly, strains of group B did not circulate in Argentina and, to our knowledge, no sequences of group B were reported in other parts of the world in 2011.

### Evolutionary Rates, Population Dynamics and Phylogeographic Analyses

**Figure 3:**
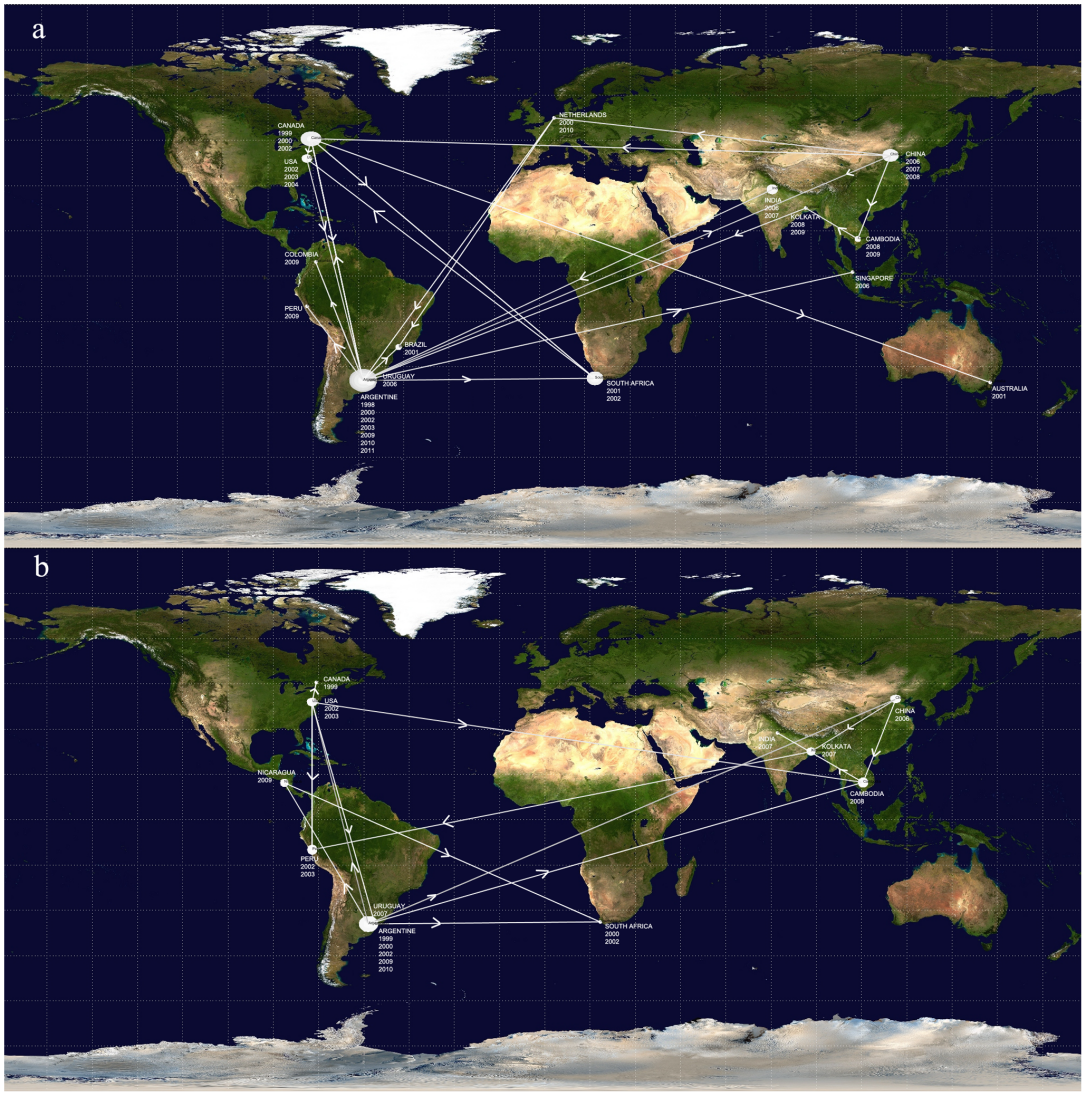
Discrete phylogeographic analysis. The date and location-annotated MCC trees based on the maximum clade credibility trees of HMPV A and B were summarized in the diffusion dynamic plots with SPREAD [18]. Lines between locations represent branches in the MCC tree along which the relevant location transition occurred. **a)** HMPV group A and **b)** HMPV group B.

The discrete phylogeographic analyses depicted by the MCC trees and summarized in the diffusion dynamic plots show how the different viral variants were globally disseminated (Figures 2 and 3). The interactive animation for group A (Video S1) also suggests that Argentine viral variants spread locally to Peru and Colombia and globally to China, where new viral variants were originated and subsequently disseminated locally (to Cambodia) and globally (to Netherlands). The diffusion dynamic analysis for group B showed spreading patterns of viral variants similar to those observed for group A. Argentine viral variants spread to China and the USA, where new viral variants were originated and disseminated locally (to India and Canada) and globally (to Peru and Cambodia). The animation also shows that Argentina could act as a source and as a spreading node of new viral variants, such as those variants disseminated to Nicaragua (Figure 3b and Video S2).

**Figure 4:**
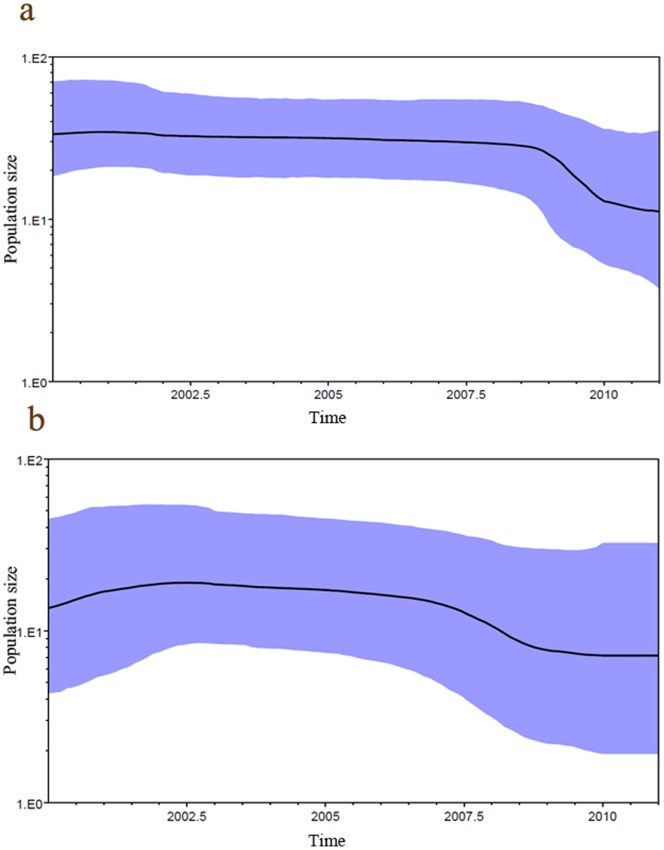
Population dynamics of genetic diversity in human metapneumovirus. Bayesian skyline plots. The horizontal axes represent time in years, the vertical axes represent the population sizes (Neτ), which are equal to the product of the effective population size (Ne) and the generation length in years (τ). The violet area gives the 95% highest probability density interval of these estimates. **a)** HMPV group A and **b)** HMPV group B.

The estimated tMRCA was 55 years (highest probability density (HPD) 95% 34 to 79) for group A and 36 years (HPD 95% 15 to 64) for group B. The Bayesian skyline plot analysis (Figures 4a and 4b) showed that the effective population size of group A remained above that of group B during the study period, although the population size of group A partially decreased after 2009.

### Molecular Characterization: Adaptive Evolutionary Analysis

The global overall divergence analysis showed that the A sequences from the G gene were more diverse than the B sequences (22.0% (SE 2.7%) and 18.4% (SE 2.5%), respectively). In addition, at the local level, the B Argentine sequences showed less divergence than the A sequences (1.7% (SE 0.4%) and 10.9% (SE 1.3%), respectively). However, the mean substitution rate observed for both groups was similar (group A: 5.16×10^−3^ nucleotide substitutions/site/year (HPD 95%: 3.77×10^−3^ to 6.59×10^−3^ nucleotide substitutions/site/year) and group B: 4.76×10^−3^ nucleotide substitutions/site/year (HPD 95%: 2.45×10^−3^ to 7.20×10^−3^nucleotide substitutions/site/year)). The mean distance calculated for the A Argentine sequences from 2009 (9.4%, SE 1.2%) was lower than that calculated for sequences from 2010 (12.4%, SE 1.6%) and 2011 (10.2%, SE 1.3%). The analysis for group B also showed less diversity during 2009 (1.4%, SE 0.4%) than during 2010 (2.3%, SE 0.5%). In addition, the nucleotide overall mean distance for both groups was lower than the amino acid overall mean distance (group A: 10.9% (SE 1.3%) vs. 15.4% (SE 2.3%), and group B: 1.7% (SE 0.4%) vs. 3.1% (SE 0.8%)).

**Figure 5:**
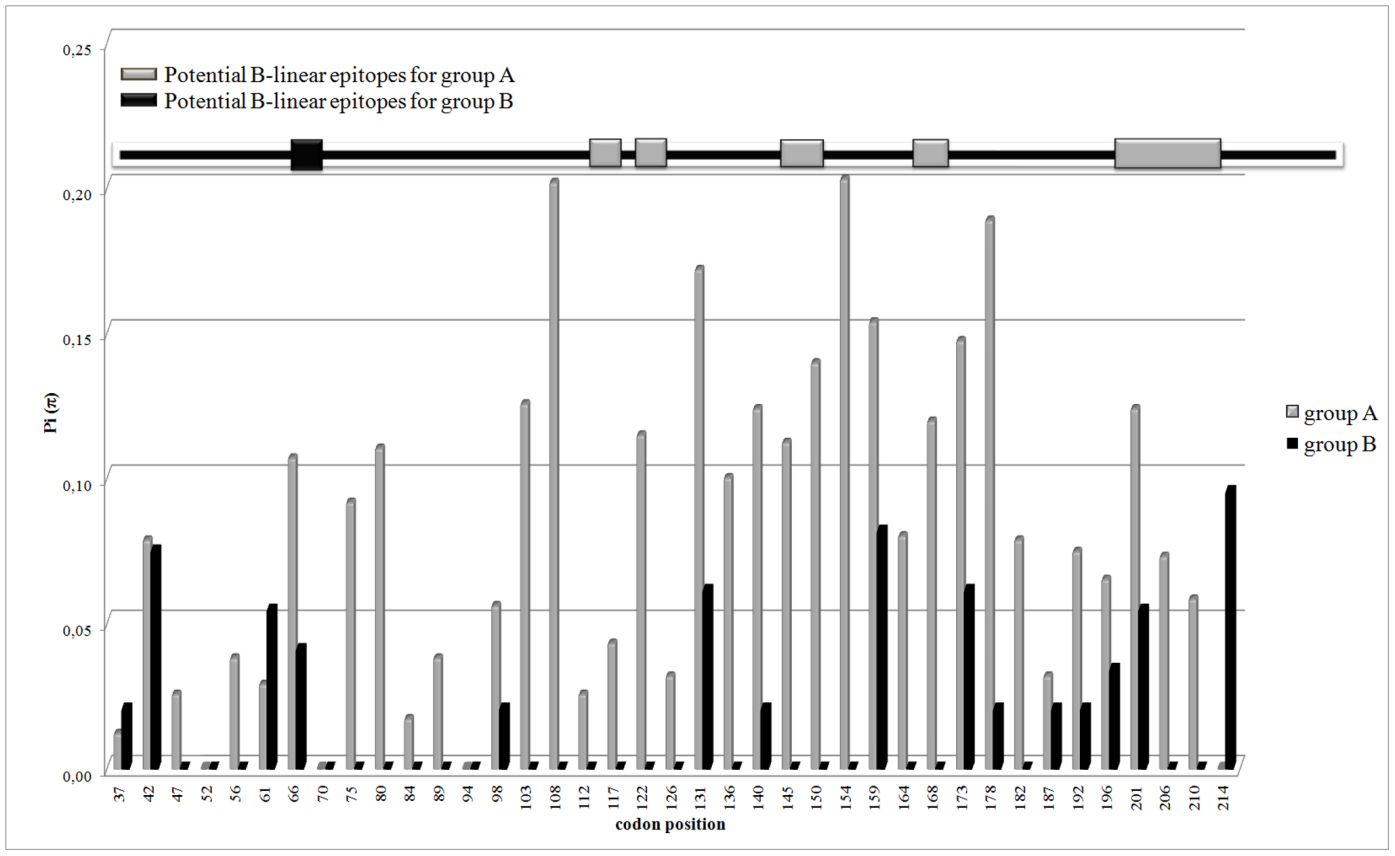
Sequence polymorphisms along the partial G protein of Argentine HMPV strains. The horizontal axe represents codons position in the G protein and the vertical axe represents sequence polymorphisms expressed as nucleotide diversity Pi (π) estimated with DNAsp [19]. Argentine sequence polymorphisms for group A are indicated with gray bars and for group B with black bars. The black square for group B, and gray squares and rectangles for group A placed in the black bar situated at the top of graph indicate the codons, around which there are the potential B-linear epitopes predicted with BCPREDS [24]. Non overlapping windows of 14 amino acids in lengths were selected to perform comparable predictions with a specificity of 75% for both analyses.

The polymorphisms of the nucleotide Argentine G sequences calculated separately for each group are shown in Figure 5. No conserved regions were found and the region between codons 131 and 178 showed the highest polymorphism. The selection pressure analysis revealed limited positive selection and abundant negative selection for group A. Indeed, only two positive sites (codons 161 and 176) were confirmed by FEL and IFEL and fifteen negative sites (codons 41, 46, 79, 110, 116, 120, 126, 143, 162, 183, 195, 197, 210, 211 and 218) were confirmed by three methods. Forty-six synonymous (*S*) and 53 non-synonymous (*N*) substitutions were found for group A and the overall ω ratio was 0.46 (p<0.1). No positive selection was found for group B but three negative sites (codons 27, 66, 158 and 230) were found and confirmed by at least two methods. Seven synonymous and 18 non-synonymous substitutions were found for group B and the overall ω ratio was 0.75 (p<0.1).

The prediction of potential B-linear epitopes for the G protein is represented in Figure 5. Most of the potential epitopes were in regions under selection pressure (mainly negative selection), and corresponded to sequence regions with low polymorphism. Codons 138, 160, 162, 195, 197 and 198 were predicted as potential O-glycosylation sites and are included in the predicted paratope binding regions. Only one epitope was predicted around codon 66 for group B, and no glycosylation was predicted at this site. The frequency of O-glycosylation was higher than that of N-glycosylation for both groups.

**Figure 6:**
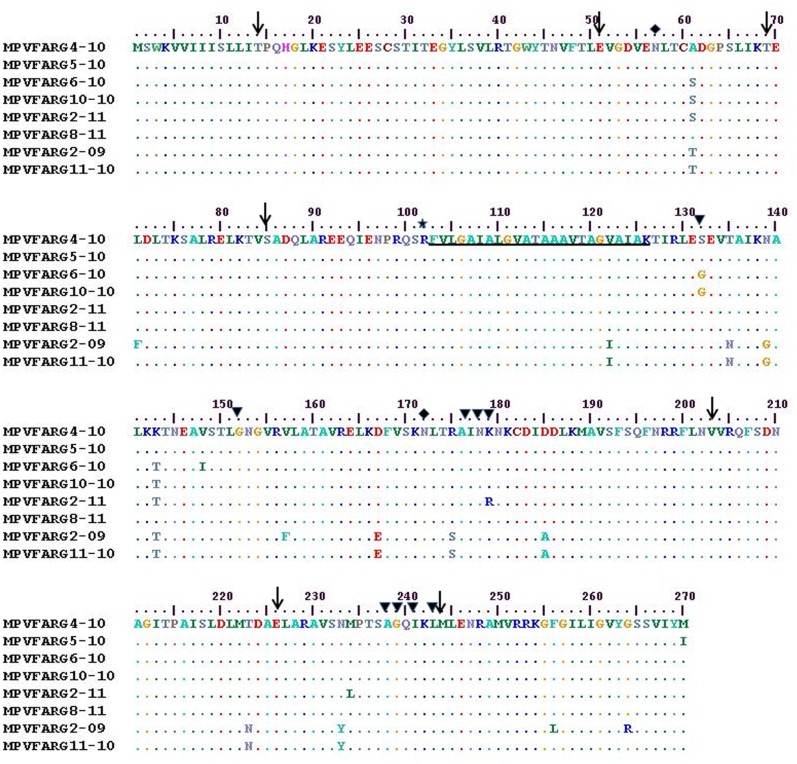
Amino acid alignment of partial F protein of Argentine HMPV strains. The sequences were aligned with CLUSTAL W [15]. Dots indicate identical residues. The first six sequences belong to group A, whereas the last two ones belong to group B. Black arrows indicate codons, around which there are the potential B-linear epitopes predicted with BCPREDS [24]. Black triangles indicate amino acids recognized by previously reported MAbs [38]. Black diamonds indicate potential N-glycosylation sites predicted with N-Glycosite [22]. The fusion peptide is underlined, and the protease cleavage site is indicated with a black star. Numbers indicate the amino acid position in the primary F open reading frame.

In contrast with that observed for the G gene, the overall mean distance of the F gene nucleotide was lower than the amino acid overall mean distance (22.9% (SE 3.7%) for nucleotides and 2.7% (SE 0.6%) for amino acids). Only negative selected sites (codons 11, 35, 83, 102, 132, 157, 164, 191, 218 and 236) were found; the overall ω ratio was 0.05 (p<0.1). The region between codons 134 and 162 showed the highest polymorphism, whereas the fusion peptide and the protease cleavage site remained conserved in all Argentine strains. Eleven potential O-glycosylation sites and only two potential N-glycosylation sites were predicted for the F gene (Figure 6).

Seven potential B-linear epitopes around codons 14, 51, 69, 85, 203, 226 and 244 were predicted for the F gene (Figure 6). None of the predicted epitopes corresponded to sites under selection pressure. Two non-synonymous substitutions were found in regions that were previously described in monoclonal antibodies (MAb)-resistant mutants (MARMs) generated against MAb 1025 (K179R for ARG2@11) and MAb 757 (S132G for ARG6 and ARG10 from 2010). The region recognized by MAbs 234 and 338 (between codons 238 and 245) remained conserved in all Argentine strains [25].

## Discussion

Since its discovery in 2001 [1], HMPV has become relevant as a respiratory pathogen in children under one year of age, having been reported as the second etiologic agent after HRSV in this age group [26]. Incidence values in China from January 2006 to December 2009 were of 9.1% [27], and those in Greece from June 2010 to June 2011 were of 3.7% [28]. A frequency of 14.7% was reported in Brazil from March 2008 to February 2009, regardless of the severity of ALRI [29]. In this study, we report frequencies as high as 21.57% in 2009. Since we analyzed only moderate and severe cases, these high frequency values demonstrate that HMPV is a significant etiologic agent in these cases. It is noteworthy the high frequency of HMPV found during the 2009 Influenza H1N1 pandemic. The reason of the high frequency might be the result of both the increase in surveillance of acute lower respiratory infections during the 2009 pandemic which continued in 2010 and the type of patients analyzed (hospitalized and mostly under one year of age), as was previously described in Brazil [29].

The phylogenetic analyses showed cocirculation of genotypes A2 and B2, as was reported in other Latin American countries such as Peru, and Brazil [29], [30]. However, in contrast with previous reports [31], we found no changes in dominance of one group over the other (A and B), but the preponderance of genotype A2 during the study period. Although we observed no alternation of phylogenetic lineages at the level of genotypes or groups (defined by genetic distances ≥15%) between years, we observed alternation at the level of phylogenetic sublineages (defined by genetic distances ≤3.4%) [1].

Galiano *et al*. have reported cocirculation of genotypes A1 and B1/A2 and B1 in Argentina in previous years [32]. No A1 and B1 strains were detected in our study and A1 has not been detected worldwide since 2004, supporting the idea that old lineages have been replaced by emerging genetic lineages [33]–[35].

Some researchers have reported no clinical differences between HMPV groups (A and B) [31], while others have found that greater severity of the illness is associated with group A [36]. Although we performed no analysis of association between genotypes and severity, genotype A2 was the genotype most frequently found in pneumonia cases or high-risk patients. However, the circulation of other genotypes cannot be rule out, and further studies are needed to analyze the strains that could be circulating in an outbreak from mild moderate and severe ALRI cases. The preponderance of genotype A2 over genotype B2 in 2009 and 2010 and the circulation of genotype A2 in 2011 only, in agreement with previous studies such as those reported by Li *et al* in China and García *et al* in Peru [7], [30], suggest that genotype A2 has higher fitness than genotype B2. This hypothesis could be supported by higher values of effective population size and genetic distance of A2 over B2. Taking into account that the effective population size could be understood as a genetic diversity measure, the results previously exposed suggest the potential ability of local and global adaptation of genotype A2. The immune response could be less effective or may develop with insufficient speed to respond against virus with high diversity [37], such as those from genotype A2.

According to Holmes, the central tenet of the phylodynamics approach is that specific epidemiological processes, particularly rates of population growth and decline, the extent of population subdivision, and the strength and form of natural selection, are written into gene sequences and can be recovered using phylogenetic techniques [37]. Consequently, the branching structure of virus phylogenies provides a unique insight and temporal of the dynamics of viral populations that allow describing the transmission chains through phylogeographic analysis [38]. The phylogeographic analyses described in the present study allow inferring that some ancestor strains of several countries could act as a source of new viral variants that subsequently spread to other countries.

The transmission chains of viral variants described for HMPV through phylogeographic analysis have shown that its migratory events occur both globally and locally, in contrast to that found for other RNA viruses, such as dengue, in which migratory events occur mainly between neighboring countries [39]. These different migration features can be explained considering the mechanisms of transmission of both viruses. The spread of dengue, which is an arbovirus, is constrained by the presence of its vector in a given geographical region, while HMPV, which is transmitted by the respiratory route, has no geographical restrictions and can travel globally harbored in hosts.

The selection pressure analysis of the F protein showed that although it has regions with polymorphisms and mutations at sites recognized by monoclonal antibodies previously described [25], it has vast structural and functional constraints, denoted by both the fusion peptide and protease cleavage site conserved regions and numerous negative selection pressure sites, together with a very low overall ω ratio. Here we predict potential B-linear epitopes, which were conserved in all the sequences analyzed. Although an *in vitro* and *in vivo* analysis is necessary to confirm the functionality of these epitopes, they may contribute to the general knowledge of the F protein as candidate for future prophylactic therapies or vaccine development. In this regard, Ulbrandt *et al*. reported the production of two monoclonal antibodies, MAbs 234 and 338, against the HMPV F protein that neutralize all HMPV genotypes *in vitro* and *in vivo*, with neutralizing capacities comparable with those of palivizumab for HRSV [40]. Here, we report the conservation of the binding sites against these MAbs (234 and 338) in all the Argentine strains. Most of the strains reported here were the etiologic agents of severe ALRI in infants and high-risk patients, supporting the idea that the humanization of both antibodies may result in viable clinical candidates able to prevent HMPV infections in high-risk patients around the world.

The understanding of the evolutionary mechanisms and chains of viral transmission in the population and a comprehensive molecular characterization of the circulating viral variants are the first steps towards deciding which candidate proteins will be part of a vaccine or preventive therapy targets in the future.

## Supporting Information

Table S1 **Background information of each sequence used in this work.**
(XLS)

Video S1 **Interactive virtual global animation for HMPV group A.** The KML file can be played by Google Earth (http: earth.google.com).
(KML)

Video S2 **Interactive virtual global animation for HMPV group B.** The KML file can be played by Google Earth (http: earth.google.com).
(KML)
